# Opioid-Free Anesthesia in a Young Female Undergoing Emergency Videolaparoscopic Appendectomy: A Case Report

**DOI:** 10.7759/cureus.68196

**Published:** 2024-08-30

**Authors:** Stefano Barbaro, Pierdomenico Carone, Laura Lanotte, Ester Scapini, Michele Debitonto

**Affiliations:** 1 Anesthesia, Resuscitation and Pain Therapy, Ospedale Monsignor Dimiccoli, Barletta, ITA; 2 Medical Oncology, Ospedale Monsignor Dimiccoli, Barletta, ITA

**Keywords:** pain management, opioids, opioid free anesthesia, general anesthesia, anesthesia

## Abstract

Opioids are one of the main classes of drugs used in anesthesia, both for the intraoperative and postoperative period, for a multitude of surgical procedures. In recent years, anesthetic techniques have been developed that reduce or avoid the use of opioids and their side effects. One of these is opioid-free anesthesia, which does not involve the use of opioid drugs in the intraoperative period. In this case report, we used this anesthetic technique for an emergency videolaparoscopic appendectomy for a young woman in a state of sepsis with a history of previous surgery for the removal of a voluminous ovarian cyst.

## Introduction

Already in 1990, the concept of reducing the use of opioid drugs arose, so Kehlet and Dahl introduced the concept of multimodal anesthesia with the aim of reducing the well-known side effects of opioid drugs, historically the cornerstone of general anesthesia [1,2]. Opioid-free anesthesia (OFA) is an anesthetic practice wherein opioid drugs are not administered in the intraoperative interval. Opioids are associated with numerous side effects such as drowsiness, dizziness, constipation, postoperative nausea and vomiting (PONV), respiratory depression, itchiness, urine retention, brief muscle rigidity, weak pharyngeal musculature, hyperalgesia, and opioid tolerance [3]. Multi-modal analgesia provides for the use of sympathetic medications and non-opioid analgesics that can reduce or prevent the use of opioids in the postoperative step [4]. Nonopioid analgesics, which can be given intravenously in the intraoperative period, are alpha-2 agonists, beta-blockers, gabapentinoids, magnesium, lidocaine, ketamine, and dexamethasone [3]. It was demonstrated that all these medicines, with their analgesic effects when administered simultaneously, change the pathophysiologic responsible process in nociception, but currently, the combination of these drugs is less used than the use of opioid drugs due to the presence of more scientific literature [5]. The α2-adrenergic agonists (clonidine and dexmedetomidine) promote analgesia, anti-hyperalgesia, sedation, anxiolysis, anti-hemesis, and sympatholysis, so they are thought to provide hemodynamic stabilization, an effect that opioids supply as well [6]. Intravenously administered lidocaine has several beneficial effects related to antinociceptive and antihyperalgesic properties as well as anti-inflammatory properties. The combined mechanisms of effect result in clinical benefits: analgesia with morphine-sparing effect, morphine-sparing secondary reduction in PONV, earlier return to transit, faster rehabilitation, and reduced duration of hospital stay [6]. Chia et al. [7] demonstrated that patients in the high-dose group had higher pain intensity at four and eight hours postoperatively, more fentanyl consumption, and a greater incidence of emesis in the postoperative period of 16 hours than those in the low-dose group (P < 0.05). Our patient had an Apfel score of 3, with a 24-hour risk of PONV estimated at 61%, with a history of PONV due to a previous gynecological surgery for ovarian cyst removal by videolaparoscopy, which is why the patient was suggested to use this alternative anesthetic technique.

## Case presentation

**Figure 1:**
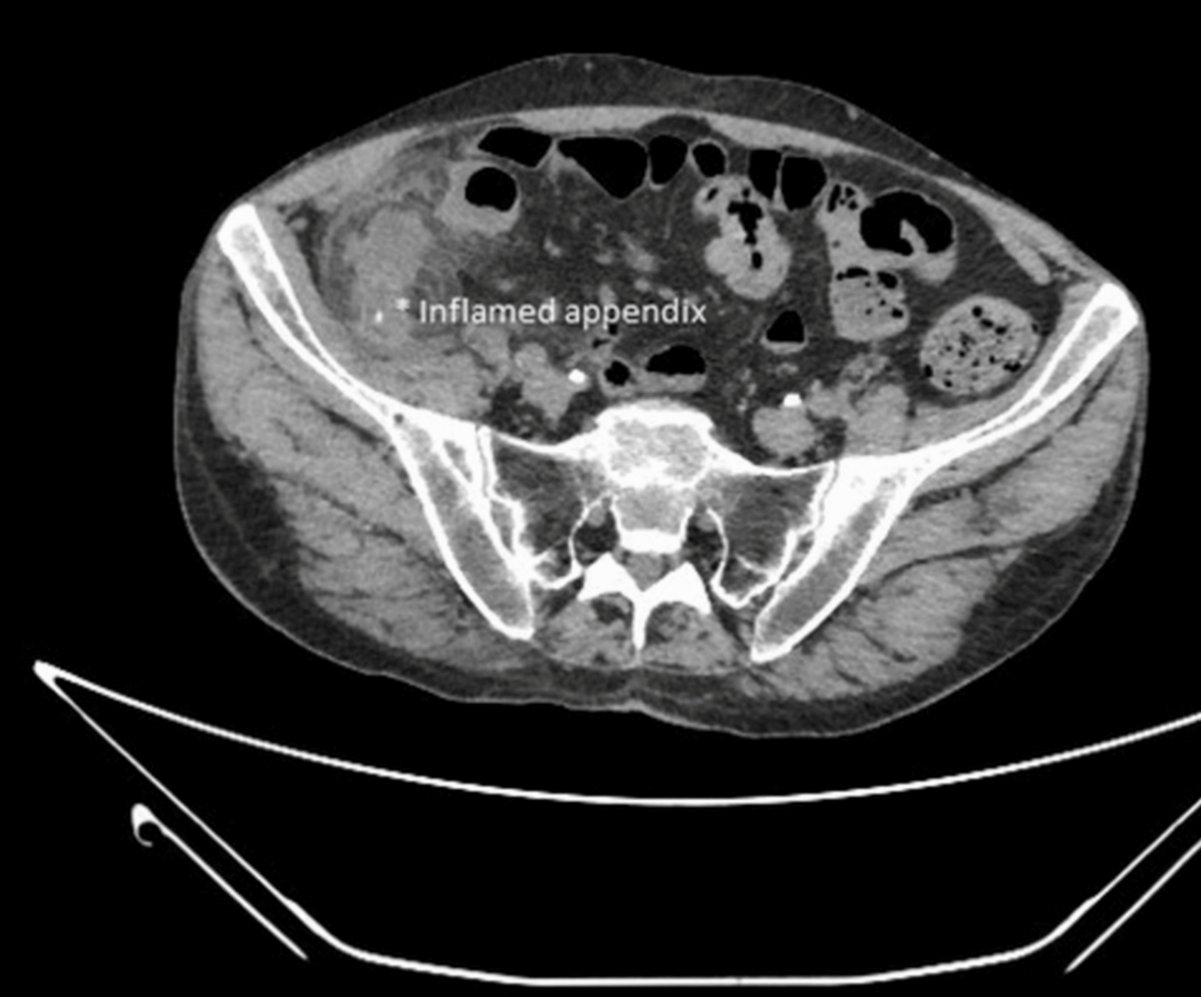
Acute appendicitis on CT scan The presence of pronounced dilatation (approximately 2 cm in diameter) and uneven thickening of the walls of the vermiform appendix indicates the presence of brief endoluminal calcific formations, likely attributed to coprolites.

A 26-year-old female in excellent health with a body mass index (BMI) of 29 kg/m^2^ reported abdominal tenderness and fever for about 24 hours, despite the initiation of antibiotic therapy with oral amoxicillin and nonsteroidal anti-inflammatory drugs. Due to the persistence of symptoms, she decided to go to the local hospital. At first observation, the patient presented with epigastric pain followed by brief nausea, vomiting, and anorexia, with pain moving to the right lower abdominal quadrant. It increased with coughing and movement. On objective examination, important was direct and rebound tenderness in the right lower quadrant, localized at McBurney's point (junction of the middle and lateral thirds of the line joining the umbilicus with the anterosuperior iliac spine). Additional signs that aroused suspicion of appendicitis were pain felt in the right lower quadrant with palpation of the left lower quadrant (Rovsing's sign). She underwent emergency blood tests with evidence of white blood cell elevation (15,000 units per milliliter of blood) and computerized axial tomography (CAT), which confirmed the clinical diagnosis of acute appendicitis (Figure 1). The Mantrels score was 10 out of 10. This diagnosis was followed by an evaluation by the general surgeon who studied the clinical case to place an indication for surgery in agreement with the patient.

Preoperatively, the patient was alert and conscious, with mild pyrexia (37.8 degrees centigrade), and stable hemodynamics (blood pressure 130/60 millimeters of mercury, heart rate of 90 beats per minute). She was placed on continuous hemodynamic monitoring: electrocardiographic record (ECG), heart rate, measurement of non-invasive blood pressure every five minutes, and oxygen saturation. Two 18-gauge cannula needles were placed in the operating room, and ceftriaxone 2 gms, dexamethasone 0.1 mg/kg, and 1 g intravenous paracetamol were administered. General anesthesia was induced with those agents: midazolam 0.04 mg/kg, lignocaine 1 mg/kg, propofol 2 mg/kg, and rocuronium bromide 0.8 mg/kg. After endotracheal intubation, ketamine 0.5 mg/kg was given, and continuous intravenous administration of lignocaine 2 mg/kg/hr and magnesium sulfate 2 g/hr was started. The patient was mechanically ventilated with ventilation mode pressure-controlled ventilation-volume guaranteed (PVC-VG), with a tidal volume of 8 mL/kg and a gas mixture of 40% oxygen and 60% air, positive end-expiratory pressure (PEEP) 4 centimeters of water (cmH2O). General anesthesia was maintained with sevoflurane, with 0.8-1 minimum alveolar concentration (MAC). After the surgery’s end, continuous intravenous infusion with lidocaine was stopped, and 30 mg of ketorolac and 4 mg of ondansetron were given intravenously. The residual neuromuscular blockade was antagonized with sugammadex 2 mg/kg, and when the patient had regular breathing, endotracheal extubation was performed. The surgery lasted a total of 40 minutes. The patient woke up without nausea, vomiting, and respiratory depression. No side effects such as dizziness, itchiness, urine retention, or brief muscle rigidity were noted; no changes in blood tests, and no changes in electrocardiography were observed in patients during the postoperative period. Postoperative analgesia provided paracetamol intravenously every eight hours and ketorolac 30 mg every 12 hours for two days. In the first hour postoperatively, the patient rated her pain as 2 on the Visual Analogue Scale (VAS). At 6, 12, 24, and 48 hours after the operation, she reported no pain (VAS score 0).

## Discussion

OFA is an anesthetic technique that does not involve opioid drugs in the intraoperative period. This technique is being implemented to decrease or eliminate adverse events related to the use of opioid drugs, which to date appear to be the mainstay of anesthesia. Progress has been made in OFA, especially in some surgical specialties such as gynecology, bariatric surgery, and orthopedics. This was also possible thanks to the greater spread and progression of neuroaxial and locoregional anesthesia techniques. The OFA involves the administration of a variety of drugs, but there are no unified protocols for drug combinations because generally opioid drugs, despite their numerous side effects, ensure adequate anesthetic coverage in the intraoperative period and hemodynamic stability. To date, appendectomy operations are performed under videolaparoscopy in the majority of cases and therefore under general anesthesia or neuraxial/locoregional anesthesia based on patient preference [8]. This is to have greater comfort for the patient and the surgeon himself. The incidence of PONV has become about 5-10% with the use of opioids and standard use of anti-emetics and adequate intra-operative pain relief, including laparoscopic interventions [9]. In daily clinical practice, we have a series of antiemetic drugs that are associated with the administration of opioid drugs, including metoclopramide, ondansetron, and dexamethasone, drugs that can be administered individually or simultaneously [10]. The adoption of anesthesia free from opioid drugs involves the use of alternative drugs such as ketamine, α2-agonists, lidocaine, magnesium, antisteroid drugs, and, where indicated, neuraxial and loco-regional anesthesia, without the use of opioid drugs [10]. Another consideration is that the use of opioid drugs in the intraoperative period leads patients to require the administration of greater quantities of opioid drugs in the postoperative period, and this leads us to introduce the concept of opioid-induced hyperalgesia and opioid tolerance [11]. However, not all patients can undergo OFA.

The main contraindications to this anesthetic technique are allergy to local anesthetics, cardiac rhythm disorders, critical coronary stenosis, cerebrovascular disorders, hypovolemic shock and hemodynamically unstable patients, acute blood loss, use of beta-blocking drugs, and an American Society of Anesthesiologists score (ASA) of 4 [12]. In our study, we examined a young woman using the Apfel score, which takes into account gender, smoking status, history of motion sickness or PONV, and use of postoperative opioids [13]. Until recently, OFA was used only for certain types of surgery, such as breast surgery, gynecological surgery, bariatric surgery, and coronary surgery [10]. It was seen that the use of OFA for some surgical interventions improved the performance relating to the Enhanced Recovery After Surgery (ERAS) protocol, a protocol that aims to reduce hospitalization times and costs per single hospital admission with a view to improving patient outcomes [14]. OFA is successful and quite safe, as it provides good postoperative analgesia while sparing opioid drugs such as morphine or tramadol, which help facilitate a faster recovery [15]. More than 200 million surgeries are performed worldwide each year; the concept of OFA could be the next goal for operating patient safety on a global scale [16,17].

## Conclusions

This case report uniquely demonstrates the application of OFA in an emergency surgery setting, specifically during a videolaparoscopic appendectomy, which is typically performed under opioid-based anesthesia. The patient, who had a high risk of PONV, was successfully managed using a comprehensive multimodal non-opioid analgesic approach. This alternative approach to anesthesia is expected to grow in the future to overcome the limitations of opioids and to improve patients's surgical outcomes. However, particular attention must be paid to the selection of the patient to undergo OFA, and standardization in OFA protocols would be of considerable importance.
